# Wild edible plants traditionally collected and used in southern Yemen

**DOI:** 10.1186/s13002-021-00475-8

**Published:** 2021-08-09

**Authors:** Mohamed Al-Fatimi

**Affiliations:** Department of Pharmacognosy, Faculty of Pharmacy, Aden University, P.O. Box 5411, Maalla, Aden, Yemen

**Keywords:** Traditional knowledge, Wild edible plant, Ethnobotany

## Abstract

**Background:**

The local wild edible plants (WEPs) are still used traditionally in the rural communities in Yemen, but this traditional knowledge is still undocumented and has been never reported before. Therefore, this study is the first ethnobotanical survey on WEPs conducted in Yemen.

**Methods:**

The study is based on two field surveys made in two periods 1988–1992 and 2014–2016 to document the wild plants used as edible by local indigenous peoples in 23 districts belonged to five governorates, in southern Yemen. Information data were collected by oral face-to-face interviews from 250 informants. Citations numbers were calculated for each species.

**Results:**

A total of 58 plant species belonged to 37 genera and 21 families are reported as wild edible plants consumed in southern Yemen. Apocynaceae was the dominant plant family with 18 species followed by Asteraceae (6) and Malvaceae (5). The most widely used edible parts are stem, leaf and fruit with more than 17 species for each. Herbs were reported as the most important sources (31 species), followed by shrubs (16) and trees (9). Most of reported wild edibles (48 species parts) are consumed in raw form; only 12 of them are cooked. Seven wild edible plants were collected in dry season, 16 species throughout the year and 38 in rainy season. In this study, 58 wild plants were reported for the first time as food in Yemen. Comparing the southern Yemeni findings to those from other world countries, 12 of them are new WEPs eaten only in southern Yemen, while 46 species are shared in the use in different world countries practically in East Africa and Arab countries.

**Conclusions:**

The results data reflect the strong relationship between the local peoples and the local WEPs as potential sources insure food security. The traditional use of these WEPs is attributed to food shortage, nutritional values and local cultural tradition. The study is of great importance in preserving the traditional and knowledge heritage from being lost due to the risks of time, war and immigration.

## Introduction

Collection of wild plants was the ancient source of food for human besides hunting; however, this wild source has been continued also after the emergence of agriculture and animal husbandry. Ethnic peoples have kept in their memory the traditional knowledge of wild food plants as heritage and transmitted it orally through generations. The relationship between human and plant is considered an ecological balance system since ancient times to preserve living organisms in the earth. Therefore, the “ethnobotany” studies the relationship between humans and plants. It aims to survey and to document different local wild plants using by ethnic groups for therapy, nutrition and economic proposes [1]. In developed countries, in Europe, wild edible plant (WEP) is considered to be an iconic ecosystem factor [2]. Many ethnobotanical researches have resulted the importance of the wild edible in saving people during famine, drought and war in different developing and developed world countries [3, 4].

Even there is now modern agriculture technology, the local people in Yemen still have strong relationship to the WEP. Many conditions that have made an attention by local people to the wild plants as an important food include the economic crises, the unlimited war and the continuous political and tribal conflicts. Moreover, the aridity and drought are general climatic characters of the Yemeni tropical southern rural regions, due to the lack of rains during one or more years; therefore, the agriculture did not provide sufficient crops for food. In this situation, local people were dependent on consumption of local agricultural crops until the eighteenth century. Southern Yemen including the study area has a large geographical region and wide diversity in mountains, deserts, plains and islands [5–7]. It has a rich diversity of plants, especially in some of the islands and the inner depth of the country [7].

On the other hand, along with its importance as edible, modern science is proving to be important sources for disease resistance [5]. In ancient Arab-Islam medicine, *Al-Razi* (865–910 C.E.) identified for the first time the relationship between food and medicine. He wrote a famous book about the “benefits of the foods and avoid their undesired effect”; that has the fact that “your medicine is in your food.” Therefore, we can indicate that *Al-Razi* was the father of the health nutrition or nutraceutical science.

The phytochemical contents of many WEPs have previously been investigated focused on their nutritional and antioxidant constituents, which affect human nutrition and health [8]. Generally, WEP contains two metabolites groups: a) the primary metabolites, which are essential agents for the organism life, include carbohydrates, fats, protein and mineral elements and b) the secondary metabolites including antioxidant agents mostly polyphenols and vitamins that protect plants. Compared to the cultivated food plants, the wild food plants are the ideal and richer natural sources for the inorganic metabolites (minerals) for balance of human and animal health. The high content of the mineral elements gives the important differences between the wild and cultivated food plants.

The available published studies on the WEPs in Arab countries are rare, except the neighboring Oman where numerous various WEPs have been reported [9]. In contrast, there is high interest in the regional African countries such as Ethiopia [4, 10, 11] focusing on studies of the WEPs.

**Fig. 1:**
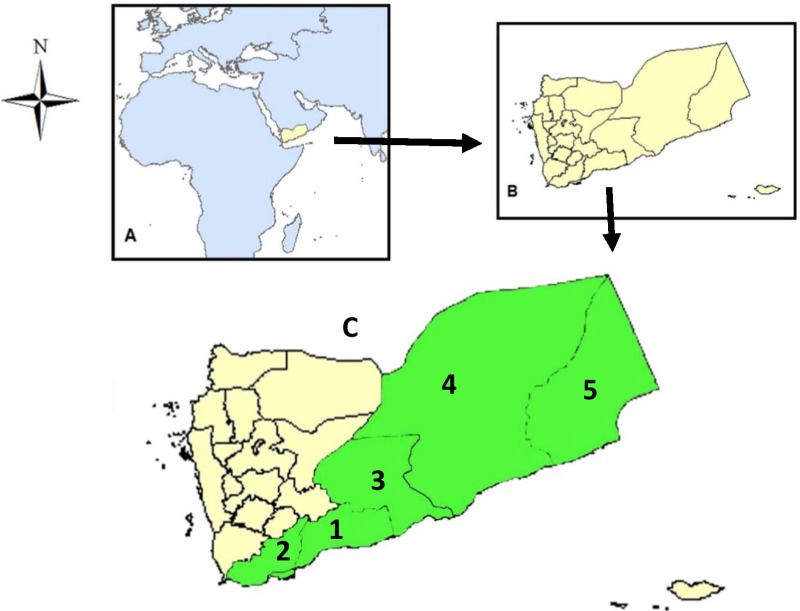
**a** Map of Yemen position in the world,** b** map of Yemen, **c** with green color, map of southern Yemen including studies governorates no **1–5**, Abyan, Lahj-Al Dahlee, Shabwa, Hadhramout and Al-Mahrah, respectively

Moreover, there are no previous reports about the traditional knowledge on WEPs plants neither in southern Yemen nor in whole Yemen except for Soqotra Island, which were previously reported by Miller [6]. Therefore, the current study presents for the first time the WEPs of southern Yemen as well as for whole Yemen (Fig. 1). This will be establishing the basis of the documented data of the traditional knowledge of WEPs for Yemen and Arabian Peninsula.

The aims of the current study are: (1) documentation of the wild plants using as food for human in southern Yemen and (2) identification of the new species used as food by comparison with the reported WEPs with data of other traditional systems in neighboring, regional and world countries.

## Materials and methods

### Study area

The study region is the southern part of Yemen that is located in southwest of the Arabian Peninsula. It is bordered by only two Arab neighboring countries: Saudi Arabia to the northeast and Oman to the east. The west part of southern Yemen is bounded by Gulf of Aden and Red Sea. The southern part is bordered by the Gulf Aden and Arabian Sea, with approximately 1580 km (Fig. 1).

The terrain of the study region is high (1500–2200 m) in the northwestern part and low (300–1500 m) in the middle part. Valleys (Wadis) spread between costal stripe and highlands. The desert of the Rub al Khali occupies in the northeastern part of the study region [7, 12]. The climate of southern Yemen is dominated by subtropical climate. The temperature is an average of 25–30 °C, but it slight changes with seasons and terrain. Rains fall in winter (February to April) and in summer (July–October); however, the rainfall is very poor in many years. Therefore, the region is dominated by drought, arid and semi-arid conditions [5, 12].

The plants distributed in the study region can be divided according to the terrain into plants growing in valleys (Wadis) and low plains, and plants growing on high plateaus, mountain slopes and mountain tops [12]. This diverse nature creates a distinctive flora of southern Arabia with many indigenous and endemic plants [5].

The study region comprises Abyan, Lahj-Al-Dhalee, Shabwa, Hadhramout and Al-Mahrah governorates with 23 districts in southern Yemen (Fig. 1, Tables 1, 2). In Yemen, including the study region in the southern Yemen, the local people are divided into Arab tribes, but they are united in historical, ethnic, and linguistic origins, customs and religion (Islam); this extends to include tribes deployed in neighboring Arab countries (Table 1). The ethnic data in the study regions are shown in Table 1.

**Table 1:** Major data of the study region and informants

Governorates	Areas (villages and urban areas)	Topography, climate, altitude	Arabic ethnic Divided into tribes; language (L	Informant Age (years) A: 12–19; B: 20–49; C: 50–85 Total (n = 250) Male: Female (total 190: 60) Literate: Illiterate: (55:195)
Abyan	Lawdar, Mukeiras, Modyah, Al-Wadhee, Gischan	Hills, mountains, Subtropical climate 500- 2200 m	Al- Awadhel, Dathani, and Fadhli tribes; L: Arabic	A: 15, B: 20, C: 35; Total 70 Male: Female = 55: 15 Literate: Illiterate = 12:58
Lahj and Al-Dhalee	Halymeen, Radfan, Yafee-Yaher Al-Dhalee	Coastal area, Hills, mountains, Subtropical climate 200- 2000 m	Abadel, Shaaibi, Jafaii, Subeehi tribes L: Arabic	A: 15, B: 20, C: 15; Total: 50 Male:Female = 35:15 Literate:Illiterate = 11:39
Shabwa	Rudhum Haban, Alrowdhah, Ataq, Gardan, Maifaah	Hills, mountains, Subtropical climate, 200- 1500 m	Awalaqi tribes; L: Arabic	A: 8, B: 15 C: 20; Total: 45 Male:Female = 35: 10 Literate:Illiterate = 10:35
Hadramout	Al-Mukala Sheher, Dawaan, Tream Syoon-Shibam	Coastal area, Sahara, Hills, mountains, 100- 1000 m Subtropical climate	Katheri, Qaeedi tribes; L: Arabic	A: 15, B: 20, C: 30; Total: 65 Male:Female 48: 17 Literate:Illiterate = 17:48
Al-Mahrah	Qeedhah, Haoof	Coastal area, Sahara, Hills, mountains, Subtropical Climate 100–800 m	Mahari tribes; L: Arabic and Mahri	A: 5, B: 5, C: 10; Total: 20 Male:Female = 17:3 Literate:Illiterate = 5: 15

**Table 2:** Ethnobotanical data of wild edible plants used by local people in southern Yemen

Botanical Data: Plant species name (Plant family), Voucher numbers	Local name of species in (Citation area)	Life form/season	Edible part: Consumed modes; taste; (Number of citations, n = 250)	Similar use in other countries [References]*
*Acacia gerrardii* Benth (Fabaceae), SA 83	Tree: Taleh (1e), Gum: Samaq (1e)	Tree/dry	Gum: chewed and eaten or drink fresh; gum dries as globules from on the stems, it dries while inside is still liquid. It is broken to drink the liquid gum; mucilage; (40)	Uganda [14]
*Acacia nilotica* (L.) Delile (Fabaceae), SA 89	Tree: Qaradh (1a-1e,2a-2c,3), Gum: Samaq (1a, 3), Samaa, Sumaa (1b,1c,1d)	Tree/dry	Gum: chewed and eaten fresh; same as above mucilage; (150)	Sudan [15]
*Acacia senegal* (L.) Willd. (Fabaceae), SA 85	Qutad (1b,1c,1d), Gum: Samaa (1b,1c,1d)	Tree/dry	Gum: chewed and eaten fresh; Same as above mucilage; (60)	Africa [10]
***Aloe lanata*** T.A.McCoy & Lavranos (Xanthorrhoeaceae), SA 64	Shrub: Anded (2), Flower: Alhab (2)	Shrub/dry	Flower: fresh cooked with bread or reis; sweet; (40)	-
*Amaranthus blitum* L (Amaranthaceae) SA 70	Ladah (1b-1d), Annass (1c,1d), Gana (1e, 3a), Dhadah (1e,4)	Herb/rainy	Leaf: fresh cooked boiled with little salts; eaten with or without bread mild sweet (160)	Ethiopia [16] Himalaya [17] Pakistan [3]
*Amaranthus graecizans* L. (Amaranthaceae) SA 71	Dhadah (1a,1e,4), Ladah (1b), Annass (1b,1c), Gana (1e,3a)	Herb/rainy	Leaf: fresh, cooked boiled wilt little salts; eaten with or without bread; mild sweet; (160)	Oman [9]
*Amaranthus spinosus* L. (Amaranthaceae) SA 72	Dhadah (1a,4)	Herb/rainy	Leaf: fresh, cooked boiled with little salts; eaten with or without bread; mild sweet (50)	Himalaya [17] Angola [18] Libya [19]
***Anisotes trisulcus*** (Forssk.) Nees (Acanthaceae) SA 51	Mudhadh (1,3), Sheri (2a), Masis (2b)	Shrub/rainy	Flower nectar: fresh suck and drink the nectar of the stigma; sweet (120)	-
*Apteranthes tuberculata* (N.E. Br.) Meve & Liede (Apocynaceae) SA 40	Khusmaa (1a,2b)	Herb/rainy	Young stem: cut and eaten fresh; bitter (90)	Pakistan [20]
*Boerhavia elegans* Choisy (Nyctaginaceae) SA 30	Hidwan (4)	Herb/rainy	Leaf: eaten fresh with or without bread; bitter; (50)	Iran [21]
*Caralluma subulata* (Forssk.) Decne. (Apocynaceae) SA 42	Shawrer (2a,2c)	Herb/rainy	Young stem: cut and eaten fresh; bitter; (30)	Namibia [22]
*Ceropegia bulbosa* Roxb. (Apocynaceae), SA 95	Alat-khalah (1b,1c,1d), Roob (5)	Herb/rainy	Young stem: cut and eaten fresh; mild sour (70)	Oman [9]
			Root (tuber); eaten fresh; sweet (50)	
*Cissus rotundifolia* Vahl (Vitaceae), SA 21	Alfaq (1a,1b,1c), Alfaq (2a), Alfaq (3b, 3c) Hadel (2a,2b),	Climber/all year	Young leaf: fresh boiled cooked with salt; eaten with or without bread or reis mild sour; (165)	Yemen [23]
*Cleome gynandra* L. (Cleomaceae), SA 22	Raab (1b,1c)	Shrub/rainy	Leaf: cooked by boiling with salts, mostly in mixture with *Cissus rotundifolia* leaf; bitter; (60)	Ethiopia [4]
*Coccinia grandis* (L.) Voigt (Cucurbitaceae), SA 25	Maqd, (1a,1e,2c), Mad (1b,1c,1d)	Climber/rainy	Fruit: eaten fresh ripe; mild sweet (60)	Himalaya [17] Ethiopia [4] Saudi Arabia [24]
*Corchorus tridens* L. (Malvaceae), SA 26	Weakeh (1b), Legen (1b,1c), Quddah (2b)	Herb/rainy	Leaf: fresh cooked; mild bitter (80)	South Africa [25]
*Corchorus trilocularis* L. (Malvaceae), SA 27	Legen, Weakeh (1)	Herb/rainy	Leaf: fresh cooked; mild bitter (60)	Ethiopia [26]
*Crepis rueppellii* Sch. Bip. (Asteraceae), SA 12	Kaanan (1c,1d,3), Hamham (5)	Herb/rainy	Root excluding coat: eaten fresh; sweet (50)	Oman [9]
***Cynanchum vanlessenii*** (Lavranos) Goyder (Apocynaceae), SA 44	Qalab (1a)	Shrub/rainy	Flower: eaten fresh; mild sweet; (5)	-
*Cynanchum viminale* (L.) L. (Apocynaceae), SA 41	Arnaq (1b,1c,1d), Khal (1c), Radhaa (2a,2c), Bidar (3)	Shrub/rainy	Young stem: eaten fresh; mild sour (150)	Oman [9] South Africa [27]
***Cynanchum viminale*** subsp. ***stipitaceum* (**Forssk.) Meve & Liede (Apocynaceae), SA 60	Radhaa (2a,2c), Milab (3)	Shrub/rainy	Young stem: eaten fresh; Sour (30)	-
*Cynanchum viminale* subsp. *suberosum* (Meve & Liede) Goyder (Apocynaceae), SA 43	Zab-Imbaeer (1b,1c,1d,1e)	Shrub/rainy	Young stem: eaten fresh; sour (65)	Oman [9]
***Desmidorchis awdelianus*** (Deflers) Meve & Liede (Apocynaceae), SA 45	Uruz (1b,1c), Kuaaur, (1b,1c,1d,1e,3d), Muqrer, (2a,2b), Kuaath (3f)	Herb/rainy	Young stem: eaten fresh; mild bitter; (80)	-
*Desmidorchis flavus* (N. E. Br.) Meve & Liede (Apocynaceae), SA 46	Uruz (1b,1c,1d), Dhaba, Dhagohom (5)	Herb/rainy	Young stem: eaten fresh; mild bitter; (85)	Oman [28]
***Desmidorchis lavrani* (**Rauh & Wertel) Meve & Liede (Apocynaceae), SA 47	Uruz (1b,1c,1d), Obar (1b,1c,1d,2a,3e)	Herb/rainy	Young stem: eaten fresh; mild bitter; (60)	-
*Ficus ingens* (Mig.) Mig. (Moraceae), SA 09	Dharef (3)	Tree/all year	Fruit: eaten fresh ripe; mild sour; (30)	Uganda [29]
*Ficus palmata* Forssk. (Moraceae), SA 10	Tree: Areen (1a), Fruit: Balas (1a,2a,b)	Tree/all year	Fruit: eaten fresh ripe; mild sour (120)	Ethiopia [4] Pakistan [3] Saudi Arabia [30]
*Ficus sycomorus* L. (Moraceae), SA 11	Saqam (1,2), Suqmi (2a,2b), Saqum (3)	Tree/all year	Fruit excocarp: eaten fresh ripe; sweet; (20)	Ethiopia [4] Sudan [31] Palestine [32]
*Ficus vasta* Forssk. (Moraceae), SA 13	Tauluq (1,2), Tauluq (3) Tiq (5)	Tree/all year	Fruit excocarp: eaten fresh ripe; sour; (15)	Ethiopia [4]
*Gladiolus candidus* (Rendle) Goldblatt (Iridaceae), SA 14	Bedhah (5)	Herb/dry	Corm: stir-fried cooked; sweet (20)	Oman [9]
*Glossonema varians* (Stocks) Benth. ex Hook.f. (Apocynaceae), SA 15	Qumredh (1c), Kobash (1d)	Herb/rainy	Fruit: Fresh unripe eaten; very sweet; (10)	Oman [9]
*Grewia erythraea* Schweinf* (*Malvaceae), SA 80	Shrub: Schohudt (1b,1c,1d,1e), Fruit: Hunqass (1b,1c,1d,1e)	Shrub/ all year	Fruit (ripe, red): eaten fresh; sweet (50)	Soqotra [6] Oman [9] Angola [18] Ethiopia [16]
*Grewia mollis* Juss. (Malvaceae), SA 81	Nashem (2a)	Shrub/ all year	Young fruit: eaten fresh; Sweet; (20)	Ethiopia [33]
*Grewia tenax* (Forssk.) Fiori (Malvaceae), SA 59	Schohudt (1a,1b,1c,1d,1e), Khedar (2a)	Shrub/ all year	Young fruit: eaten fresh; sweet; (60)	Ethiopia [16] Sudan [31]
*Hydnora abyssinica* A.Br. (Aristolochiaceae), SA 03	Nabeekh (1b), Trateef (1c,1e), Fateekh (1c,3a,3b), Twacheen, Kuaarer, Twhoot (2b), Ftookh (3c), Imlokh (5)	Herb/rainy	Fleshy Flower: eaten fresh or stir-fried cooked; fresh: astringent; mild sour, aroma; cooked: mild sweet; (160)	Yemen [34] Uganda [29]
*Lactuca serriola* L. (Asteraceae), SA 04	Lessan- imthawr (1b,1c,1e)	Herb/rainy	Leaf: eaten fresh without or with bread; mild bitter; (60)	Turkey [35] Libya [19]
*Launaea intybacea* (Jacq.) Beauverd (Asteraceae), SA 05	Hawa (1a), Lessan- imthawr (1b,1c,1e), Hawa (2a), Lessan-albaqarah (4)	Herb/rainy	Leaf: eaten fresh without or with bread; mild bitter (60)	Ethiopia [11]
*Launaea procumbens* (Roxb.) Ramayya & Rajagopal (Asteraceae), SA 06	Hawa (1a,2a)	Herb/rainy	Leaf: eaten fresh without or with bread; mild bitter; (20)	Ethiopia [4]
***Lavandula pubescens*** Decne. (Lamiaceae), SA 07	Feheh (2b)	Shrub/dry	Leaf: eaten fresh with bread; mild bitter, aromatic; (20)	–
***Monolluma hexagona*** (Lavranos) Meve & Liede (Apocynaceae) SA 52	Mequrezh (1a, 1e), Uruz (1b,1c)	Herb/rainy	Young stem: eaten fresh; mild bitter; (60)	–
*Monolluma quadrangula (*Forssk.) Plowes (Apocynaceae), SA 48	Mequrezh (1a, 1e), Uruz (urzh) (1b,1c,1d,2b), Mulaauzah (2a), Qarnat-adhabia (3a), Hwimadhah (3c), Dhaba, Dhagohom (5)	Herb/rainy	Young stem: eaten fresh; mild bitter; (180)	Oman [9]
***Monolluma solenophora*** (Lavranos) Meve & Liede (Apocynaceae), SA 49	Khusmaa (1a,2b), Uruz (1b,1c)	Herb/rainy	Young stem: eaten fresh; mild bitter; (50)	–
*Opuntia ficus-indica* (L.) Mill. (Cactaceae), SA 53	Tarung (1a), Rangah, Teen (2b)	Shrub/ all year	Fruit: eaten fresh peeled; sweet; (120)	Ethiopia [4] Libya [19] Algeria [36]
***Orbea wissmannii* (**O. Schwartz) Bruyns var. *wissmannii* (Apocynaceae), SA 50	Khusmaa (1a), Uruz (1b,1c,1d)	Herb/rainy	Young stem: eaten fresh; mild bitter; (60)	–
*Plantago major* L. (Plantaginaceae), SA 54	Lessan-imthaur (1b,1c,1d,1e)	Herb/rainy	Leaf: eaten fresh; Bitter; (25)	Spain [37] Libya 19] Lebanon [38]
*Portulaca oleracea* L. (Portulacaceae), SA 55	Dhuras (1a,1b,1c,1d), Sungulah (2a), Qalqalah (2b), Rubidta (4), Hamdhieh (5)	Herb/rainy	Leaf, stem: eaten fresh as salad with bread or without; cooked with *Corchorus tridens;* mild sour; (180)	Turkey [35] Oman [9] Libya [19] Egypt [39] Palestine [32] Lebanon [38] Morocco [39] Jordan [40] Sudan [41] Saudi Arabia [42] Somalia [43] Iraq [44]
*Portulaca quadrifida* L. (Portulacaceae), SA 56	Dhuras (1d)	Herb/rainy	Leaf, stem: eaten fresh or cooked; mild sour; [49]	Ethiopia [11]
*Raphionacme velutina* Schltr. (Apocynaceae), SA 86	Qurs-bathilan (1b,1c)	Herb/rainy	Root (tuber); eaten fresh; Sweet; (20)	South Africa [27]
***Reichardia tingitana*** (L.) Roth (Asteraceae), SA 85	Khasoor (2b,2c), Hawa (2b)	Herb/rainy	Leaf, stem: eaten fresh with bread; mild bitter; (20)	-
*Rhytidocaulon macrolobum* Lavr. subsp. *macrolobum* (Apocynaceae), SA 87	Quarran (1b,1c,1d)	Herb/rainy	Young stem: chewed and eaten fresh; mild bitter; (70)	Tropical Africa [45]
*Rhytidocaulon tortum* (N.E. Br.) M.G. Gilbert (Apocynaceae), SA 88	Quarran (1b,1c,1d)	Herb/rainy	Young stem: chewed and eaten fresh; mild bitter; (70)	Tropical Africa [45]
***Rumex nervosus*** Vahl (Polygonaceae), SA 16	Uthrub (1b)	Shrub/ all year	Flower nectar: fresh sucked; mild sweet; (10)	
	Uthrub (1a,1e)		Leaf: chewed and eaten fresh; mild bitter; (50)	–
*Sageretia thea* (Osbeck) M.C. Johnst. (Rhamnaceae), SA 89	Niem (1a)	Shrub/ all year	Fruit: ripe fruit, eaten fresh; sweet; (20)	Oman [28]
*Salvadora persica* L. (Salvadoraceae), SA 90	Shub: Rak (1c), Fruit: Mard (1c)	Shrub/ all year	Fruit: ripe fruit, eaten fresh; sweet; (10)	Oman [9]
*Senna italica* Mill. (Fabaceae), SA 91	Ushruq (2a,3)	Shrub/dry	Fruit: eaten fresh; bitter; (50)	Oman [9]
*Sonchus oleraceus* (L.) L (Asteraceae), SA 92	Lessan- imthawr (1b,1c), Lessan Albuqri (2b), Lessan- athawr (3), Lessan al-baqarah (4)	Herb/rainy	Leaf: eaten fresh without or with bread; mild bitter; (90)	Turkey [35] Egypt [39] Tunisia [46]
*Ziziphus leucodermis* (Baker) O. Schwartz (Rhamnaceae), SA 94	Elb (3), Labdhi, Habedh (4), Dhood (5)	Tree/all year	Fruit: eaten fresh or dried; sweet; (50)	Oman [9]
*Ziziphus spina-christi* (L.) Desf. (Rhamnaceae), SA 93	Tree: Elb (1,2,3,4), Dhaood (5), Fruit: Daoom (1,2,3,4), Girem (5)	Tree/all year	Ripe fruit excluding kern: eaten fresh or dried; Sweet; (250)	Soqotra [6] Angola [18] Ethiopia [26] Oman [9] Libya [19] Saudi Arabia [47] Egypt [39] Sudan [31] Palestine [32] Tunisia [48] Morocco [49]
	Seed: Agarah (1a,1b,1c,1d), Seed excluding coat: Fersoos, Ferquoos, (1a,1b,1c,1d)		Seeds excluding coat: chewed and eaten fresh; Oily; (50)	Ethiopia [4] Sudan [31]

Citation area, where the WEP is collected: *1–5* The names of the governorates, 1-Abyan governorate, 1a-Mukeiras, 1b-Lawdar, 1c-Modyah, 1d-Al-Wadhee, 1e-Gischan, 2-Lahj and Al-Dhalee governorates, 2a-Radfan-Halymeen, 2b-Yafee (Yaher), 2c-Al-Dhalee, 3-Shabwa governorate, 3a-Gardan, 3b-Ataq, 3b-Alrowdhah, 3c-Haban, 3d-Saaeed, 3d-Nasab, 3e-Maifaah, 3f-Arma, 4-Hadhramout governorate, Syoon, Dawaan, Al-Mukalla, Sheher, 5-Al-Mahrah governorate, Qeedhah, Haoof

Agricultural products in southern Yemen focus on sorghum, maize, millet, wheat and barley. The local economy depends on agriculture and livestock. The production of the agriculture is low; therefore, local people still depend on the collection of the WEPs to use as security food and to use as products for marketing. On the other hand, the cultural data of the local medicinal plants in a selected part in southern Yemen were previously studies by the author [5].

### Historical data

Southern Yemen is a region historically linked to Arabian Peninsula, the Gulf of Aden and the Arabian Sea. Several centuries BC, Southern Yemen was famous for the production of frankincense tree (*Boswellia* sp.), myrrh, aloe and henna, which were transported by camels through the “incense road” in the deserts of Al-Mahra, Hadhramout and Shabwa to the north of Arabian Peninsula and around the world. These plants products have been used as food, medicine and for religious customs.

At that time southern Yemen was famous for its historical port of Qana in Shabwa on the Arabian Sea, this port was a famous and important link for the transport of plant products from Soqotra Island, East Asia and Africa to Europe across the desert.

### Data collection

Two ethnobotanical surveys were conducted in two periods at 1988–1992 and 2014–2016 to document the wild plants used as edible by local indigenous peoples in southern Yemen. Information data were collected by the author through oral face-to-face interviews among 250 Soqotri informants; most of them were adult and elderly. Ethnobotanical data that were primarily aimed to document are: local names of the plants, growth form of plants, edible parts, time of collection (season) and consumed modes and preparations. Some care was taken to document the local names of each species in different localities and the local names of the used parts or exudate products obtained from the wild plants.

The taste is an important organoleptic property used to determinate a crude drug or food. Therefore, each reported wild edible part was examined to document the taste property. Most used parts of the reported local WEPs were tested by the author himself. The characteristics tastes of WEPs are presented in Table 2.

### Local informants and demographical data

The local informants were grouped into 58 children and young (12–19 years), 192 adults (20–49) and 110 elderlies (50–85 years); mostly informants were adult and elderlies (84%). The informants were villager, farmer, shepherds, students and housewives. Most informants were illiterate (78%), but many of them were literate with primary and secondary school (Table 1). In each study region, the informants were selected with help of the local elderly people and the farmers and shepherds who work and move between the plains and mountains all over the study region and therefore have a strong relationship with nature and a close knowledge of plants. Most of them who have been inquired from live in the same study region and do not leave except when necessary. The elderly over 70 years have never left the area and have a strong and solid relationship with nature and plants. All informants have the same Arabic ethnic roots. They are divided into tribes with characteristics properties that distinguish them from others, and they all speak one single Arabic language. There are slight differences in dialect in Arabic speaks, which is understandable to all in different regions. All informants were interviewed by the author in Arabic language.

### Plant identification and plant material

The taxonomic identifications were carried out in Pharmacognosy Department, Aden University. Most of the collected plants specimens were identified using references of botanical data on Yemeni flora [5]. Voucher specimen in the form of herbarium or dried parts of plants or even photographs were deposited at Pharmacognosy department, Aden University, and in Al-Fatimi’s Herbarium. Acceptance taxonomic names of species were done using “The Plant List” (www.theplantlist.org) and for Apocynaceae species were done based on Meve and Liede [13].

### Data analysis

For each wild plant species, the citation number was calculated to express the number of informants who cited a specific wild edible plant.

### Comparative studies on literature review

Data for comparative study were collected from the published literatures using electronic databases including PubMed, Google Scholar, Scopus and Science Direct, and other Internets sources, and published books related to WEP. The obtained local data were compared with data from studies conducted in neighboring and regional countries, using these terms "ethnobotany," "wild edible plants," "wild food plants," "Arab countries" or related terms. Similar uses of reported WEPs were reviewed and cited, for neighboring countries Arabian, African and Asian countries. This aims to identify the new local wild plants that are reported as food for the first time.

## Results

### Local names of plants

**Fig. 2:**
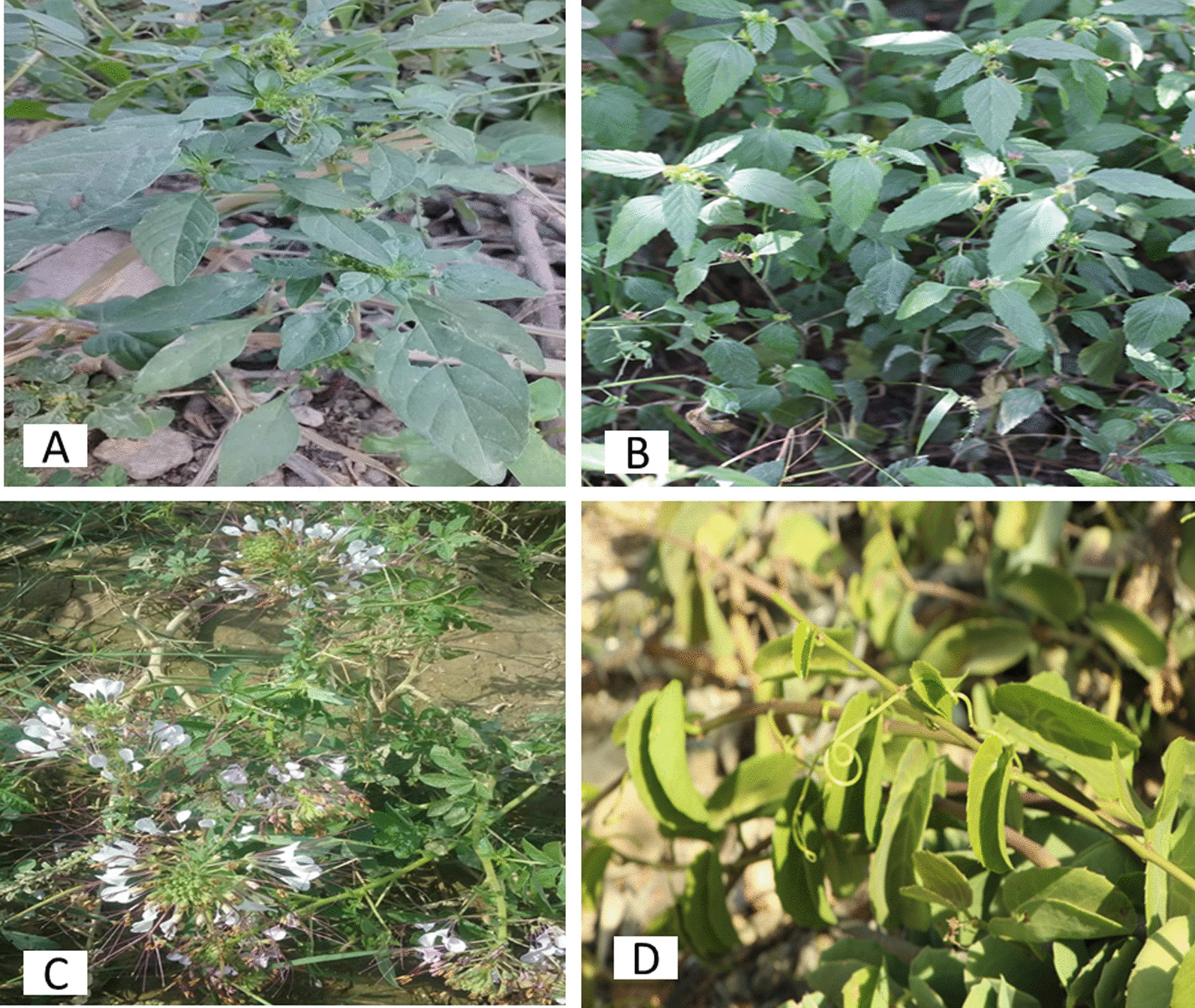
Most used local wild edible leaves in southern Yemen: botanical name (local name), (citation number). **a** *Amaranthus blitum* (*ladah, annass*), (160). **b** *Corchorus tridens* (*weakeh*) (80). **c** *Cleome gynandra* (*raab*), (60). **d** *Cissus rotundifolia* (*alfraq*), (165). (Photos made by the author)

**Fig. 3:**
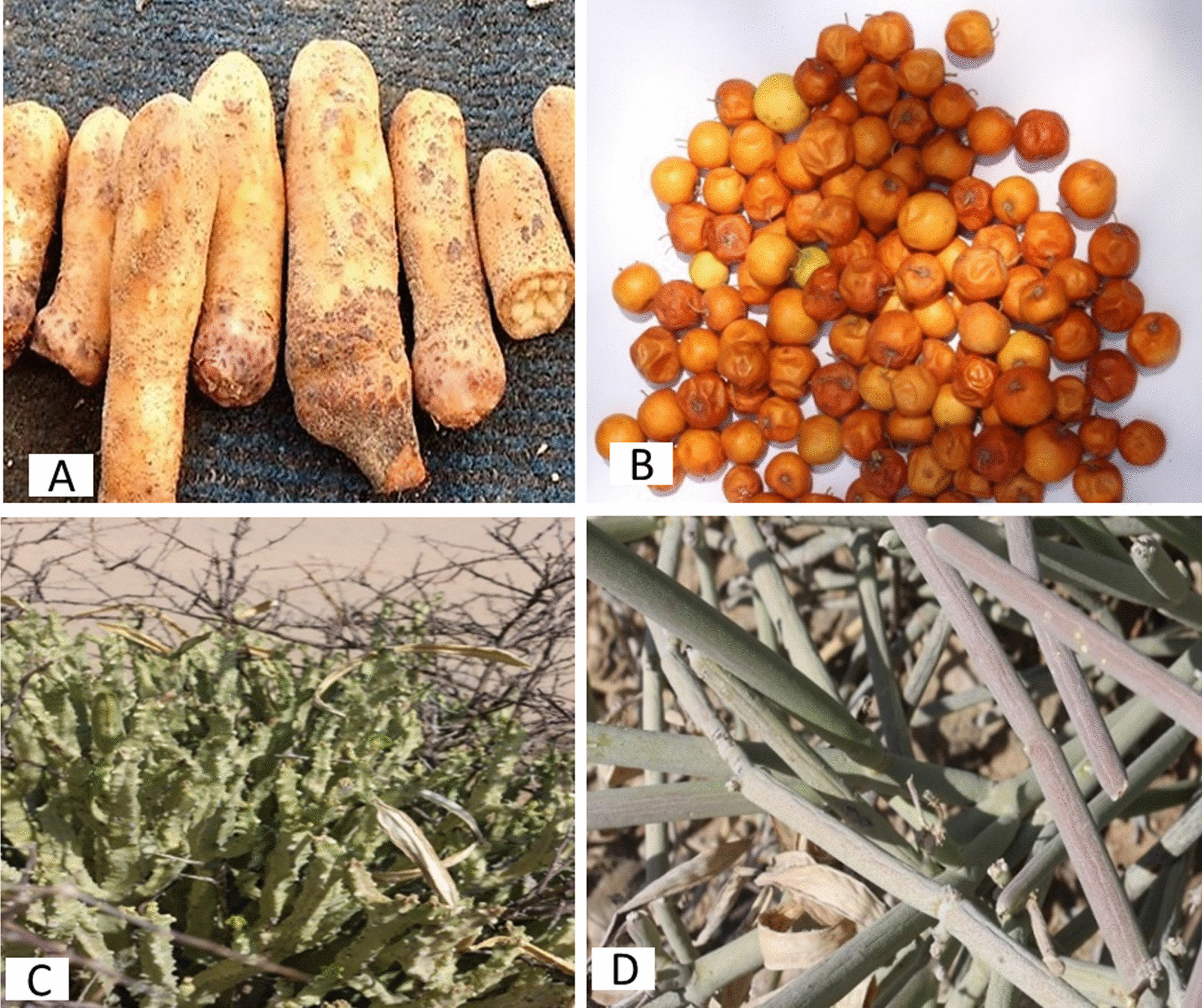
Some highest cited local wild edible plants in Southern Yemen. Botanical name (local name), used part (citation number). **a** *Hydnora abyssinica* (*nabeekh*), flower (160). **b** *Ziziphus spina-christi* (*Damoon*), fruit (250). **c** *Monolluma quadrangula* (*mequrezh, uruz*), stem (180). **d** *Cynanchum viminale* (*arnaq*), stem (150). (Photos by the author)

The indigenous people of southern Yemen are capable for naming and classifying the plants, which are used as food (Table 2). They can instantly differentiate between plants with similar morphology and give them different names. They give local names not only for the wild plant species but also for their edible parts and exudates of plants. For instance, the name of the plant species *Acacia nilotica* is *qaradh*, while the name of its edible gum is *samaa* (Table 2). Moreover, different edible parts of single plant have different local names: for example, the species *Ziziphus spina-chisti*; its tree is named *elb*, its edible fruit is named *daoom*, and the seed endosperm is named *fersoos* or *ferquoos* (Table 2). On the other hand, different species of one single genus or different subspecies of a single species, of which different parts are used, were differentiated by using different local names. For example, *arnaq* is the name of the edible stem of *Cynanchum viminale* and *alab* is the name of inedible and toxic stem of *Cynanchum viminale* subsp. undefined. These different local names help to avoid the confusion of the products, which is essential due to the toxicity of some stems of the species. The local names for each plant used in different localities (urban and villages) in southern Yemen are shown in Table 2, Figs. 2, 3.

The local people reported no specific name for prepared dish; a preparation has same local name of its used WEP.

Species names with bold font recorded as new wild edible species which not reported before in the literature, while all species reported for the first time for Yemen.

### Ethnobotanical data

Fifty-eight wild plant species were reported to be used as food sources in southern Yemen, belonging to 21 plant families and 37 genera. These traditional knowledge data obtained from two field surveys are summarized in Table 2 for use as food.

Ethnobotanical data about species, genera and families of the recorded WEPs in southern Yemen are presented in Tables 2, 3, 4. Most of edible species belong to the Apocynaceae family (18 species), followed by Asteraceae (6 species), Malvaceae (5 species), Moraceae and Fabaceae (4 species for each), Rhamnaceae and Amaranthaceae (3 species for each) and Portulacaceae (2 species). The remained 13 families presented by one species for each (Table 3). The genera presented highest species are *Cynanchum* and *Ficus* (4 species for each), *Acacia, Amaranthus, Desmidorchis, Grewia* and *Monolluma* (3 species for each) followed by *Corchorus, Launaea, Portulaca, Rhytidocaulon* and *Ziziphus* (2 species, for each). The remained twenty-five genera represented by one species for each (Table 3). This indicates the high diversity of the flora of southern Yemen regions.

**Table 3:** Botanical data of plants families, genera and species

Family name	Genera No	Species No	Number of used part
Apocynaceae	10	18	Stem: 15, Fruit: 1, Root: 1
Asteraceae	5	6	Leaf: 5, Root: 1
Malvaceae	2	5	Fruit: 3, Leaf: 2
Moraceae	1	4	Fruit: 4
Fabaceae	2	4	Fruit: 1, Stem gum: 3
Rhamnaceae	2	3	Fruit: 3
Amaranthaceae	1	3	Leaf: 3
Portulacaceae	1	2	Leaf 2
Aristolochiaceae	1	1	Leaf
Cleomaceae	1	1	Leaf
Lamiaceae	1	1	Leaf
Nyctaginaceae	1	1	Leaf
Plantaginaceae	1	1	Leaf
Vitaceae	1	1	Leaf
Xanthorrhoeaceae	1	1	Leaf juice
Cactaceae	1	1	Fruit
Cucurbitaceae	1	1	Fruit
Salvadoraceae	1	1	Fruit
Acanthaceae	1	1	Flower nectar
Polygonaceae	1	1	Flower nectar
Iridaceae	1	1	Corm
Total 21 families	37 genera	58 species	See Table 4

**Table 4:** Parts of the wild edible plants used in southern Yemen

Edible parts/exudates	Number	Percentage %
Leaf	18	26.5
Stem	18	26.5
Fruit	17	25
Flower	5	7.4
Root and corn	4	5.9
Seed	1	1.5
Gum	3	4.4
Flower nectar	2	2.9

The highest number of WEPs belongs to Apocynaceae with 18 species (Table 3). Most used parts of these species are succulent stems. Nine Apocynaceae species belonged to the most species-rich genus was *Caralluma* (divided into new genera names: *Monolluma, Orbea*, *Apteranthes*, *Desmidorchis*) with additional nine species of other genera including *Cynanchum* (4 species), *Rhytidocaulon* (2 species)*, Ceropegia, Glossonema* and *Raphionacme* (one specie per each).

### Edible parts and collection time

Sixty-three edible parts and five plant exudates are reported to obtain from 58 wild plant species. Stem and leaf were the most used parts (18 species for each), followed by fruits (17) and flowers (5) (Table 4). Moreover, products from plant parts such as plant exudates: latex, juice, resin, flower nectar and gum were reported to use by local people (Table 4).

The wild edible parts were gathered at different times during the year. Most wild edible parts (38 parts) are collected in the rainy season. While sixteen wild edible parts were collected throughout the year, 16 species parts were collected in the dry season, and only seven plant parts are collected in the dry season. *Cissus rotundifolia* Vahl leaves and *Ziziphus spina-christi* fruits are the important and most cited WEPs, which are available in whole year (Figs. 2, 3). The leaves of *Cissus rotundifolia* are still green during the year even in time of rainfall lack; therefore, these leaves were gathered and consumed during times of food shortage. Some wild food plants are found only after heavy rain, which grow quickly after rains with high-disturbed abundance on either ground such as *Amaranthus graecizans*, *Cynanchum viminale* and *Monolluma quadrangula*, or underground such as *Hydnora abyssinica*. Others wild species collected in the rainy seasons are rare and found seldom which are *Ceropegia bulbosa*, *Rhytidocaulon macrolobum*, *Rhytidocaulon tortum* and *Cynanchum viminale* subsp. *suberosum* (Table 2).

### Life forms

**Fig. 4:**
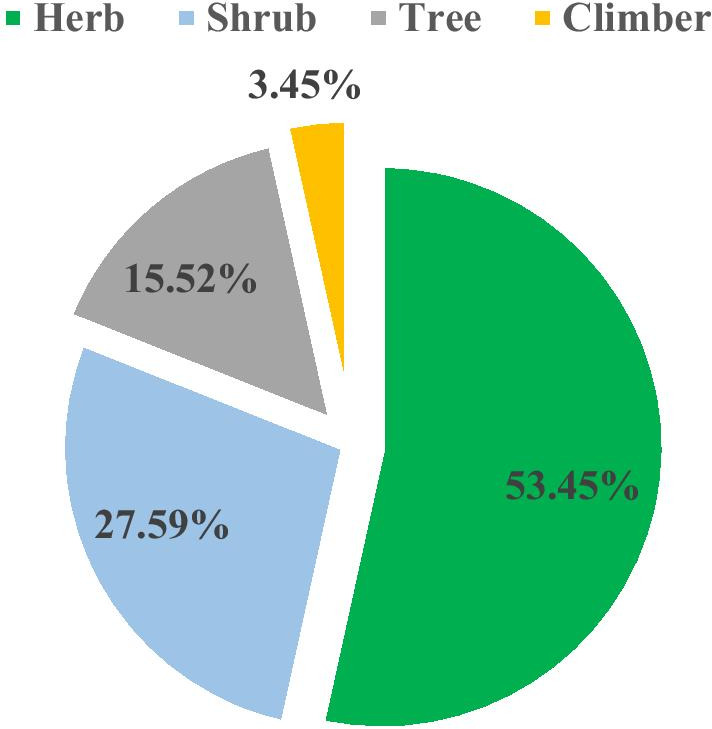
Life forms of wild edible plants in southern Yemen

Local people used WEPs with diversity in growth forms: herbs, shrubs, trees and climber. Of them 31 species are herbs that represented high percentage (53.45%) of the total species, followed by shrubs with 16 species (27.59%) and trees with 9 species (15.52%) (Fig. 4).

### Modes of consumptions and preparing of WEPs

All of the edible parts of recorded WEPs are eaten fresh and immediately after collection and are not stored except the fruits of *Ziziphus spina-christi*, which can be dried and stored that were thus of critical importance in times of edible scarcity. Even gum of the *Acacia* species, the local people like to eat it fresh semiliquid. Nineteen of total 61 reported wild edible parts are cited to eat as tender young parts. Most reported wild plants parts (46 species) are eaten fresh raw and uncooked, while only 9 edible parts are cooked (Table 5), for example, *Amaranthus graecizans*, *Cissus rotundifolia*, *Cleome gynandra* and *Corchorus tridens*, and the residue three edible parts could be eaten either raw or cooked, including *Hydnora abysinica* flower, *Portulaca oleracea* and *Portulaca quadrifida* herb (Tables 2, 5).

**Table 5:** Wild edible plants associated with different usage categories of consumed modes

Use category (58 Species)	Edible part/exudates (Species No.)	Wild edible species
Cooked (9 species)	Leaves (7)	*Amaranthus blitum, Amaranthus graecizans*, *Amaranthus spinosus, Cissus rotundifolia*, *Cleome gynandra*, *Corchorus tridens, Corchorus trilocularis*
	Corn (1)	*Gladiolus candidus,*
	Flower (1)	*Aloe lanata*
Eaten raw (46 species)	Exudate: liquid and dried Gum (3)	*Acacia gerrardii, Acacia nilotica, Acacia Senegal*
	Fruits (14)	*Coccinia grandis*, *Ficus ingens, Ficus palmata, Ficus sycomorus, Ficus vasta, Grewia erythraea, Grewia mollis, Grewia tenax, Opuntia ficus-indica, Sageretia thea, Salvadora persica, Senna italica, Ziziphus spina-christi, Ziziphus leucodermis*
	Stems (14)	*Apteranthes tuberculata, Caralluma subulata, Ceropegia bulbosa, Cynanchum viminale, Cynanchum viminale subsp. Stipitaceum, Cynanchum viminale subsp. Suberosum, Cynanchum vanlessenii, Desmidorchis awdelianus, Desmidorchis flavus, Desmidorchis lavrani, Monolluma hexagona, Monolluma quadrangula, Monolluma solenophora, Orbea wissmannii*
	Roots or corns (4)	*Glossonema varians, Raphionacme velutina,* *Rhytidocaulon macrolobum, Rhytidocaulon tortum*
	Leaves (9)	*Lactuca serriola, Launaea intybacea, Launaea, procumbens, Lavandula pubescens*, *Plantago major*, *Reichardia tingitana*, *Rumex nervosus, Sonchus oleraceus, Boerhavia elegans*
	Exudates: Flowers nectar (2)	*Anisotes trisulcus, Rumex nervosus,*
Cooked or raw eaten (3 species)	Flower (1)	*Hydnora abysinica*
	Leaf, Stem (2)	*Portulaca oleracea*, *Portulaca quadrifida*

The fresh leaves of *Amaranthus graecizans, Amaranthus blitum, Cleome gynandra, Corchorus tridens* and *Corchorus trilocularis* are collected, cleaned with water, cut into small pieces and boiled in water with a little salt for ten to fifteen minutes (Table 5). All these WEPs leaves appear in the rainy time, mostly near farmland. The cooked leaves of *Amaranthus graecizans*, *Amaranthus blitum* and *Corchorus tridens* are prepared as soft pastry food and eaten with or without bread. All these WEPs leaves have mucilage taste and are eaten only cooked with salt. *Portulaca oleracea* leaves have salty taste and can be eaten either fresh or cooked, in sauce or with yogurt and with bread or reis (Table 5). Mostly these leaves are cooked with other cultivated vegetables as sauce called "*sanonah*." Some of these plants may be cooked together such as leaves mixture of both *Portulaca oleracea* and *Corchorus trilocularis* or leaves mixture of *Cleome gynandra* leaves with *Cissus rotundifolia*.

*Cissus rotundifolia* leaves appear all year. The fresh leaves of *Cissus rotundifolia* are boiling in water with salt, where the cooked leaves are eaten as paste. In some area of southern Yemen, the leaves of *Cissus rotundifolia* can be cooked with *Cleome gynandra* leaves. In other case, the crushed leaves are mixed in water with wheat or millet flour in the boiling water to make the flour dough. In the mixing process, a woody stick from *Ziziphus spina-christi* is used. This method was mostly used in the time of crop shortage (Table 5). The uncooked leaves have a sour taste similar to vinegar; therefore, it was named as a "natural vinegar" in the Arab old manuscripts of traditional food and medicine.

**Fig. 5:**
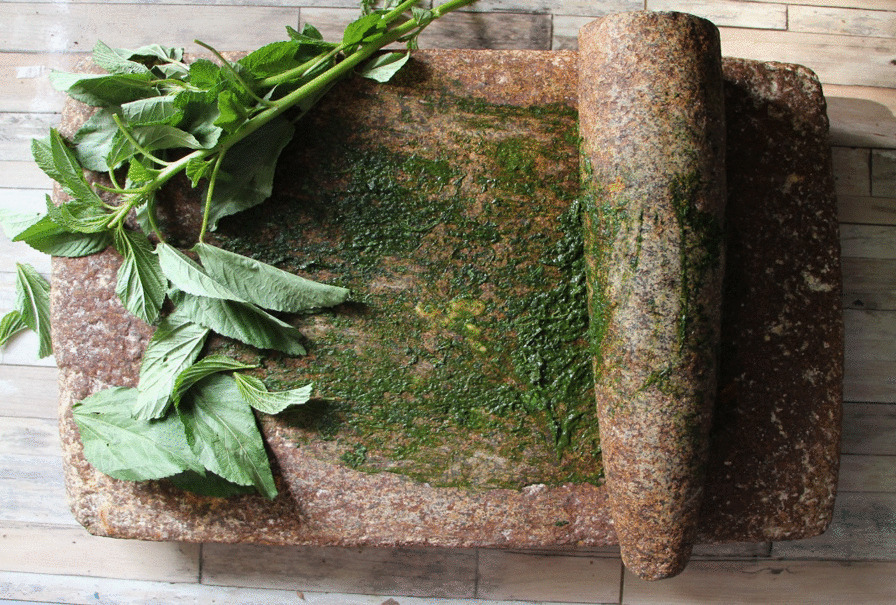
A traditional stone mill used to grind local edible wild plants

On the other hand, the edible leaves are crushed and ground on a traditional stone mill called in some areas "*merhah*" (Fig. 5); it is a crushing stone that is made by a local traditional method. Nowadays, some people use an electric mixer; however, local people prefer to use the traditional stone mill because they believe that it gives food a distinctive and delicious taste.

The flower of the parasite plant *Hydnora abysinica* is fleshy and soft part; it has aromatic smell because it contains volatile compounds that we reported in a previous study [34]. The flower is eaten fresh raw with aromatic and astringent taste. However, the local people mostly like to eat it cooked; in this case, the flowers are usually grilled on the woody coals, thus eliminating the volatile components. Therefore, the cooked flower becomes mild sour and delicious taste with a distinctive flavor.

### Traditional knowledge and citation values

Most local wild plants were reported with highest frequency to be used as edibles (Table 2, Figs. 2, 3). Thirty-four species of total 58 plants were reported by most informants based on the total number of the informants in each citation area, where edible plant grows. These include *Amaranthus graecizans, Cleome gynandra, Corchorus trilocularis*, *Desmidorchis awdelianus, Ficus palmata, Hydnora abyssinica, Monolluma quadrangula, Portulaca oleracea* and *Ziziphus spina-christi* (Tables 2, 6, Figs. 2, 3). More than 100 informants reported eleven WEPs to use frequently as edible sources. Thirty edible species were reported by more than 50 informants. Only 17 species were reported as wild edibles by equal or less than 30 informants (Table 2). There are 15 top most important WEPs cited by most informants and recommended for sustainable use (Table 6).

**Table 6:** 15 top most important WEPs cited by most informants and recommended for sustainable use

Scientific name (edible part) (Citation number)
*Acacia nilotica* (gum) (150)
*Amaranthus blitum* (leaf) (160)
*Amaranthus graecizans* (leaf) (160)
*Anisotes trisulcus* (flower nectar) (120)
*Cissus rotundifolia* (leaf) (165)
*Coccinia grandis* (fruit) (160)
*Cynanchum viminale* (stem) (150)
*Ficus palmata* (fruit) (120)
*Hydnora abyssinica* (flower) (160)
*Lactuca serriola* (leaf) (60)
*Monolluma quadrangula* (stem) (180)
*Opuntia ficus-indica* (fruit) (120)
*Orbea wissmannii wissmannii* (stem) (60)
*Portulaca oleracea* (leaf) (180)
*Ziziphus spina-christi* (fruit) (250)

### Classification of local WEPs

The WEPs are still used in southern Yemen due to their nutritional importance in the food security. Therefore, local WEPs can be classified into four groups according to their uses benefits by local peoples in the study area:

- *Wild edible plants are using during time of food shortage*: *Ziziphus spina-christi* (fruit),

*Cissus rotundifolia* (leaf) and *Monolluma quadrangula* (stem).

The edible *Cissus rotundifolia* leaves are evergreen throughout the year. It is found in abundance all over Yemen. Therefore, it is usually used by the poor people in this war in all Yemen region. *Ziziphus spina-christi* is widespread in all regions of southern Yemen; it grows mostly on the edges of agricultural areas. Its fruits are important food for the local people, that are eaten either fresh or dried. Dried fruits are sweeter than fresh fruits that can be stored for some weeks. This plant is considered as an important botanical and ecological manifestation of the region. It is used not only as a source of food, but for various popular uses, the most important of which are for shade, grazing, household appliances and for many medicinal properties [5]. Immature fruit has a slight sweet with sour taste while fully ripe fruit has a very sweet taste with slight sour taste. By the drying of the green fruits, their color becomes orange-red and they lose their acidity and their taste becomes very sweet. This is evidence that the red fruit contains a high percentage of sugars that assist to storing for a long time. On the other hand, local people like to eat the young succulent, fleshy stems of Apocynaceae WEPs growing after the rains; especially, they like to eat the upper branches newly growing in the stems of these WEPs. The stem has a sticky mucilage taste and a sweet bitterness.

- *Wild edible plants are used for home consumption by family*:

These WEPs grow with sufficient water in rainy time. Therefore, they are used as supplements to households due to their luxury and delicious nutritional values and have been cited by all informants, including leaves of *Amaranthus graecizans, Amaranthus blitum, Corchorus trilocularis, Cleome gynandra, Portulaca oleracea* and *Lactuca serriola*. Local people like to eat these edible leaves due to their sweet tastes and nutritional benefits. The fruits of *Ficus palmata* are one of the most important wild fruits that people like to eat for their delicious tastes and as home consumption. This plant often grows in high places in the region, but their presence is seasonal and limited.

- *Wild edible parts are using regular by children*: as delicious wild foods, including *Ziziphus*

*spina-christi* (fruit), *Hydnora abyssinica* (flower)*, Anisotes trisulcus* (flower nectar) and *Opuntia ficus-indica* (fruit). The traditional knowledge of WEPs among the children and young over 12 years was high and accurate. They can collect the fruits of *Ziziphus spina-christi* in big mass. *Z. spina-chisti* is one of the most popular WEPs that local people commonly eat, especially children. Moreover, the children know the growth places of *Hydnora abysinica* flowers, in the underground and their growth time, especially after the rainy period. Children like to pick the red flowers of *Anisotes trisulcus* and suck the very sweet-tasting flower nectar to get energy. *Opuntia ficus-indica* grows on the high plateaus of the southern region. Its fruits are sold in popular markets as delicious sweet fruits.

### Organoleptic characters of edible parts

The taste character of each wild edible part was tested by author or cited by informants. The results are shown in Table 2. Twenty-four wild edible parts are described with bitter taste, followed by twenty-three parts with sweet taste, eleven have sour taste and three products have mucilaginous taste. The most edible parts have palatable bitter tastes. Elder local people in the study region say, “What it is bitter (*murr*), it is a medicine (*dawa*)”. The characteristic delicious taste and the nutritional value of WEPs were a important motivation that led to protect and sustain this traditional knowledge by local people. The taste is an important organoleptic property to determinate the quality of a crude drug and food.

### Comparative study

The results of the comparative studied are shown in Tables 2, 7, 8. These include comparison of the recorded data in the study region with the ethnobotanical literature of neighboring countries, Arab countries and regional countries.

**Table 7:** Novel WEPs for southern Yemen and similar WEPs used limited in Arabic and regional countries based on the literature review

Novel WEPs for southern Yemen (12)	WEPs used in Arab countries including Yemen (14 species)	WEPs used in Yemen and in regional non-Arab countries (15 species)
*Aloe lanata* (flower) *Anisotes trisulcus* (flower) *Cynanchum vanlessenii* (flower) *Cynanchum viminale* subsp. *Stipitaceum (*stem) *Desmidorchis awdelianus* (stem) *Desmidorchis lavrani* (stem) *Monolluma hexagona* (stem) *Monolluma solenophora* (stem) *Orbea wissmannii* (stem) *Reichardia tingitana* (leaf) *Rumex nervosus* (flower) *Lavandula pubescens* (leaf)	**Oman (12 species)** *Amaranthus graecizans,* *Ceropegia bulbosa,* *Crepis rueppellii, Cynanchum viminale subsp. Suberosum, Desmidorchis flavus, Gladiolus candidus, Glossonema varians, Monolluma quadrangula, Salvadora persica,* *Senna italica,* *Sageretia thea,* *Ziziphus leucodermis* **Saudis Arabia** (2 species) *Cissus rotundifolia,* *Rumex nervosus* (leaf) **Sudan** *Acacia nilotica*	**Ethiopia (7)** *Corchorus trilocularis,* *Cleome gynandra,* *Ficus vasta,* *Grewia mollis,* *Launaea intybacea,* *Launaea procumbens, Portulaca quadrifida* **Uganda (2)** *Acacia gerrardii,* *Ficus ingens* **South Africa (2)** *Corchorus tridens, Raphionacme velutina* **Namibia (1)** *Caralluma subulata* **Pakistan (1)** *Apteranthes tuberculata* **Iran (1)** *Boerhavia elegans*

**Table 8:** Similar WEPs used in Arab region and in limited regional countries (14 WEPs)

WEP used in study area	Same WEP used in other Arab and (regional countries)
*Hydnora abyssinica*	Oman, (Uganda)
*Cynanchum viminale*	Oman, (South Africa)
*Grewia erythraea*	Oman, (Angola, Ethiopia)
*Amaranthus spinosus*	Libya, (Angola, Himalaya)
*Lactuca serriola*	Libya, (Turkey)
*Opuntia ficus-indica*	Libya, Algeria, (Ethiopia)
*Plantago major*	Libya, Lebanon, (Spain)
*Coccinia grandis*	Saudi Arabia, (Ethiopia, Himalaya)
*Ficus palmata*	Saudi Arabia, (Ethiopia, Pakistan)
*Ficus sycomorus*	Sudan, Palestine (Ethiopia)
*Grewia tenax*	Sudan, (Ethiopia)
*Sonchus oleraceus*	Egypt, Tunisia, Iraq (Turkey)
*Portulaca oleracea*	Oman, Libya, Egypt, Palestine, Lebanon, Morocco, Jordan, Sudan, Saudi Arabia, Iraq, Somalia (Turkey)
*Ziziphus spina-christi*	Oman, Libya, Saudi Arabia, Egypt, Sudan, Palestine, Tunisia, Morocco (Angola, Ethiopia)

## Discussion

### Study area

The study area was selected due to its important biodiversity and ethnobotany [5, 7] and due to lack of the documentation for the traditional knowledge of the WEPs neither in the study area nor in whole of Yemen. Therefore, this study establishes the first and fundamental step for the traditional knowledge of local wild plants using as food and nutraceuticals in Yemen and Arab countries. We observed in the study area that there is a strong relationship between local people and the environment, and therefore, they have a high traditional knowledge that transmits among indigenous communities. They could instinctively able to discover WEPs and avoid poisonous or wild inedible plants. In addition, the local people have traditional behavior to the rational use of the roots of the WEPs [5]. Moreover, most of the recorded WEPs are wide distributed across the southern Yemen.

### Botanical data

Local people can accurately determine the local wild plant species with different local names, according to their morphological characteristics. They can differentiate between species belongs to one genus to identify whether it is edible or toxic. For instance, they can distinguish between apparently very similar species of *Aloe* and *Cynanchum*.

The importance of recorded WEPs in the study area appears in their wide diversity and higher number of species (58), genera (37) and families (21). This wide diversity of WEPs provides a wide and various alternative sources of food; this supports the food security in the region.

### WEPs from Apocynaceae

Apocynaceae has the highest number of wild edible species (18 species, 31%) in the study area, due to the high abundance and diversity of this family species in the flora of southern Yemen [5, 7]. Generally, WEPs belonged to Apocynaceae were reported to have limited geographical tropical origins including the study area (southern Yemen) and some African countries. Comparing the WEPs of Apocynaceae recorded in the study region with ethnobotanical studies in other world countries, six species of Apocynaceae were reported to use as food in Oman [9] and five species in Pakistan, Namibia and South Africa (Tables 2, 7). Interestingly, seven succulent WEPs are recorded as new WEPs that are used only in southern Yemen based on the literature review. Two similar wild edible species used in southern Yemen and South Africa are *Cynanchum viminale* and *Raphionacme velutina* and two similar genera but with different species are *Orbea* and *Ceropegia* [27]. In addition, comparing WEPs of Apocynaceae in the study area (southern Yemen), there are large numbers of different non-similar WEPs of Apocynaceae reported in the ethnobotanical literature of South Africa (137 species) and in Kenya (13 species) [27, 50, 51]. This reveals that the family of Apocynaceae contains not only poisonous species, but also safe species that are edible and healthy for humans and have been used since ancient times by different ethnic peoples. It leads to indicate that the reported large number of Apocynaceae shows safe uses as food (140 Apocynaceae species for only both Yemen and South Africa are used as WEPs). Interestingly, there is a great similarity between Yemen and South Africa in the ethnobotanical uses of Apocynaceae as source foe wild edible species. The difference in the number and the diversity of Apocynaceae species are due to the different climate and terrain between the two countries.

The reason may be due to a similarity in the environment, especially the presence of both countries on the edge of an ocean with very long coasts.

We can classify the large various species of Apocynaceae according to their safety and toxicity into main two groups as inedible and edible species. The inedible Apocynaceae species contain toxic phenols, cardiac glycosides and alkaloids as major secondary metabolites; these poisonous species are important sources for isolated therapeutical drugs. The edible Apocynaceae species contain primary metabolites as major content such as carbohydrates, fat, proteins and minerals besides less content of non-toxic flavonoids and phenols as secondary metabolites. The edible Apocynaceae species have high nutritional value*s*, and some of them show benefit effect on general heath especially on the metabolism disorders as antidiabetic agent [5, 20, 52–54]. On the other hand, the uses of these edible succulent to treat diabetes were reported in our previous study [5].

The wild succulent stems of Apocynaceae are most famous specific food in the study regions. These wild species form various specific and cultural features of the flora of southern Yemen.

### Life form, used parts and collection time

Herb was rarely reported as most life form for WEPs while it is the most common growth form of WEPs in the study area, due to the high number of edible succulent stems and leaves that are mostly obtained from herb. In contrast, shrub and/or trees were reported as usually most sources for wild food in different world localities such in Uganda [29] and Ethiopia [26, 55].

On the other hand, fruits were the most frequently used parts in many world countries [18, 26, 29, 56], while in the study region, three used parts: fruit, leaf and stem were reported in this study as the most used parts. Leaf is known as the main biosynthetic and store organ for primary nutritive metabolites such as carbohydrates, fat and proteins, which are biosynthetic precursors for secondary metabolites that transfer and store in other plant organs.

The large number of edible stems is due to various number of edible non-woody succulent stems of Apocynaceae that are widely distributed in southern Yemen. These succulent stems constitute mucilage as major carbohydrate metabolism product.

Wild leaves are considered as an important source with high nutritional value in minerals, proteins and energy, for instance, the leaves of *Amaranthus viridus* and *Corchorus tridens* [52]. Ten different families are sources for the edible leaves of 18 species recoded as WEPs (Table 3), while only Apocynaceae is the most common source for the most edible stems obtained from 15 species. Rhamnaceae and Moraceae are the most common sources for the edible fruits in the study region.

The high frequently use of the fresh wild edible parts indicted that the fresh parts contain highest nutritional values than the dried parts, which may be loss their nutritional metabolites by heating. The collection time of the local WEPs is dependent on the availability of the used parts in the arid study origin. The most WEPs are found only after rainfalls. The growth of the WEP in the arid and semiarid region of southern Yemen is affected by the rain (*madtar*) and draught (*gadb, gafaf*) during the year.

These findings confirm that local wild plants are still required as an important traditional food sources for local people in the study regions, similar to other many semiarid countries [11, 18]. Edible wild plants that appear with the rainy seasons are usually important. Children and families race to collect them before they disappear after the rains. Children and young people usually race to collect them because they are one of the delicious and popular local foods and for their importance as a high source of food and energy. Plants in dry seasons are collected and stored. Many of them are sold in the market.

In the recent time, all the plants recorded in this study are used as food whether in food shortage or in normal times because they have become a cultural and usual heritage as natural sources for high nutrients and power support for general health.

### Traditional knowledge, citation number and modes of consumptions

Most reported WEPs were cited to use by all the informants in each citation area where plant was collected. Local people knew most of the reported WEPs and still use them as food. This high citation level indicates two important specific data of the local traditional knowledge: 1) the high and wide distribution and sharing of local traditional knowledge among all informants types, women, men, children, young and adult in southern Yemen and 2) the high and close connection and strong relationship between the local people and nature; this indicated through their high appreciation for the environment, plants and animals. Therefore, this traditional knowledge of WEP has always been used and requested by the local people even during the economic, political and environmental events that have affected different life sides in the country.

The importance of the local WEPs appears through the great interest of the local people in collecting these edibles after the rains fall and consuming them immediately before they disappear. Therefore, these seasonal WEPs could be used as food shortage and as distinct delicious food with high nutritional value that strengthens the health level, as the local people believe in their cultural behavior.

Many WEPs are popular even outside their natural region range because of their nutritional benefit and delicious taste, for instance, fruits of *Ziziphus spina-chisti, Ficus palmata* and *Opuntia ficus-indica*, fresh leaves of *Portulaca oleracea* and fresh stems of *Monolluma quadrangula*.

### Wild edible plants in folk markets

The WEPs with highest citations frequency have also commercial values; some of them were sold at local markets, including the fresh herbs of *Amaranthus graecizans* and *Portulaca oleracea,* fresh or dried fruits and seeds of *Ziziphus spina-christi,* fresh fruit of *Ficus palmata*, and *Opuntia ficus-indica* (L.) Mill.*,* fresh stems of *Monolluma quadrangula, Desmidorchis awdelianus* and *Orbea wissmannii* Bruyns*,* fresh flowers of *Hydnora abysinica,* and fresh or dried gum of *Acacia nilotica* (Tables 2, 4)*.* Storable wild foods are only two plant products: *Acacia nilotica* (gum) and *Ziziphus spina-christi* (fruits).

Different collected wild edible parts were found in the folk markets as entire fresh, dried parts and dried exudates. In addition, some of these WEPs face and resist the risk of extinction, due to increased collecting and marketing and due to over exploitation in the last ten years such in state of *Hydnora abysinica* and *Monolluma quadrangula*.

The local cultural tradition is an important factor that made people still use the WEPs in southern Yemen. Besides the characteristic delicious tastes of the WEPs, the local peoples called this wild edible plants as “*vitamin baldi*” which means “local rural vitamins” which are also strongly required in the countries cities. In addition, the local people faith that the WEPs consumption gives a natural powerful immunity forces “*quwah*” against infectious diseases. Therefore, indigenous people described a wild edible part as “*dawa*” which means “*drug or health food*” which can give “*quwah*” which means immunity that strengthens the general health. Compared to our previous study [5], many of the local WEPs (30 species) were reported in our previous study to have local traditional medicinal uses [5].

Therefore, the local people still keep their cultural tradition and still gather WEPs as a preferred food to imported industrial food. Various numbers of WEPs are sold in the markets as an additional source of household income.

### Comparing the traditional knowledge data over time (1988–2016)

The study in the first trip 1988–1992 was aimed at an early documentation of the traditional knowledge of plants among the local people and to preserve this information from being lost due to the influence of time and other influences factors. The local people before 25 years in the targeted study areas were still in a strong connection with nature, especially wild plants to use them as food and medicine. In the second trip in 2014–2016, the aim was to find out if there is a loss or changes in the local traditional knowledge that occurred over the past two decades.

In the first trip at 1992, all informants have similar knowledge level of WEPs even the children and young informants; it was no significant difference between the number proportion of men (48%) and women (42%). In 2016, nearly 25 years after the first survey, we found a great stability in the level of traditional knowledge of WEPs among the elderly over 60 years, especially women, mostly in isolated villages and in areas that were not reached by the movement of civil expansion and the effects of war.

On the other hand, a significant weakness in the traditional knowledge among the young people was observed. Many of them could not remember some local plants names, or they confuse local names between a plant and another. The WEPs with low citation number recorded in the first survey 1988–1992 are unknown to them or hardly remembered such as *Ceropegia bulbosa, Ficus ingens, Raphionacme velutina* and *Sageretia thea*. The Young people face difficult life due to economic crises, war tensions, lack of water in rural areas and water depletion in many mountainous areas. Although this comparison proved the survival of the knowledge heritage in the traditional uses of plants among the majority of informants, these local WEPs are important and distinctive food sources for them. Even those who immigrate to cities since thirty years ago often require these WEPs due to their distinctive taste that stucks in their memory.

However, the time is a risk factor in influencing the sustainability and survival of oral unwritten traditional knowledge across the generations. The importance of plants, their protection and conservation of the cultural, biological and environmental heritage of the study area can be incorporated into the curricula of the local primary schools. The local heritage of the WEP must be explained and teach through the school, the environmental organization and the local authority. Therefore, this early study reflects the great importance benefits in preserving the local biological wealth of the WEPs in southern Yemen as a part of human traditional knowledge.

### Comparative study

This study is the first ethnobotanical survey of WEPs in Yemen in general, and in particular, in southern Yemen. The local traditional knowledge on WEPs in southern Yemen has specific indigenous species compared to the traditional knowledge of other world localities. Comparing the WEPs recorded in this study with the ethnobotanical literature in Arab and other world countries aims to know the new species that are used only in the study area and to know the extent of knowledge spread among these countries. Generally, this comparison revealed that 46 used species were reported to use in other world countries as well as in Arab countries including neighboring countries (Oman and Saudi Arabia) and this led to identify 12 new plant species that are used only in Yemen as WEPs.

### Comparative study on similar WEPs in the regional countries

Compared to the literature review, 25 wild plants recorded in the study area were reported as wild foods with same edible parts in different Africa countries (Tables 2, 7). Of them, eight WEPs recorded in the study area in southern Yemen are found to be used only in Ethiopia; these WEPs are *Corchorus trilocularis, Cleome gynandra, Ficus vasta, Grewia mollis, Launaea intybacea, Launaea procumbens* and *Portulaca quadrifida* (Table 2). Other African countries showed low number of shared WEPs that are recorded in southern Yemen, including Uganda (2 species) *Acacia gerrardii* and *Ficus ingens*, South Africa (2 species) *Corchorus tridens* and *Raphionacme velutina* and Namibia only *Caralluma subulata* (Tables 7, 8).

Other shared WEPs recorded in the study area were reported as edibles in both African and Arabian countries; these include *Cynanchum viminale* (South Africa, Oman), *Ficus sycomorus* (Ethiopia, Sudan, Palestine), *Grewia erythraea* (Ethiopia, Angola, Oman), *Grewia tenax* (Ethiopia, Sudan), *Hydnora abyssinica* (Uganda, Oman), *Ziziphus spina-christi* (Ethiopia and eight Arabic countries) (Tables 7, 8)*.*

Based on a comparison with ethnobotanical literature, 25 edible species recorded in the study area are also found to be used in traditional knowledge in some African regions, while less WEPs number (9 species) was reported to use in some Asian regions. In addition, *Apteranthes tuberculata* was reported only in Pakistan and in *Boerhavia elegans* in Iran (Tables 2, 7, 8).

*Ziziphus spina-christi, Hydnora abyssinica, Amaranthus graecizans* and *Monolluma quadrangula* are the most cited WEPs in the study area that are also frequently used in many different neighboring and regional countries (Tables 2, 7, 8). Many shared WEPs from different geographical countries have been examined and reported as rich sources for nutritional value, proximate composition and energy [8, 11, 30, 39–41, 52].

### Comparative study on similar WEPs with neighboring countries

Comparing the WEPs recorded in this study with the ethnobotanical data of the two neighboring countries (Oman, Saudi Arabia), only 5 shared WEPs were found as edibles in Saudi Arabia, while 17 shared WEPs were reported in Oman (Tables 2, 7, 8). Of them, 12 shared WEPs are new WEPs that are used only in the study region (southern Yemen) and Oman and two WEPs are used only in Southern Yemen and Saudi Arabia (Table 7). Shared WEPs that are used in Oman and southern Yemen include *Caralluma subulata* (Forssk.) stem, *Senna italica* Mill. fruit, *Crepis rueppellii* Sch. Bip. root, *Cynanchum viminale* subsp. *suberosum* root, *Gladiolus candidus* (Rendle) Goldblatt corm and *Salvadora persica* fruit (Table 2). The large similarity in the traditional uses of WEPs in both Oman and Southern Yemen is due to the existence of a common relationship between the two localities and peoples in terms of similar flora, geographical ethnic characteristics, heritage, inherited customs, language and history.

Furthermore, two similar WEPs are used in neighboring countries but with different used parts. Fruits of *Cissus rotundifolia* are used as food in Saudi Arabia [24] and roots of *H. abyssinica* used in Oman, both instead the leaves and flowers, respectively, that are used in the study area [9].

### Comparative study on similar WEPs in Arab countries

The ethnobotanical data of WEPs in Arab countries are few and limited based on the available literature. Studies with a large survey were found for Libya [19], Jordan [40], Lebanon [38] and Palestine [32]. However, in comparison with these Arab countries, we recorded a big difference regarding different plant species used, except neighboring Oman. Therefore, there are a limited number of shared WEPs (14 species) that are reported to use in Arab countries (Tables 2, 7, 8); these include Libya (6 WEPs), Palestine, Egypt and Libya (3 for each), Morocco, Lebanon and Tunisia (2 for each) and Jordan, Algeria, Iraq and Somalia (one for each) (Tables 7, 8). In addition, eight of these WEPs were reported as edibles in other some African and Asian countries compared to the ethnobotanical literature, namely *Sonchus oleraceus* (Turkey, Egypt, Tunisia), *Lactuca serriola* (Turkey, Libya), *Amaranthus spinosus* (Angola, Himalaya, Libya), *Coccinia grandis* (Ethiopia, Himalaya, Saudi Arabia), *Ficus palmata* (Ethiopia, Pakistan, Saudi Arabia) and *Portulaca oleracea* (Turkey, ten Arab countries) (Tables 2, 7, 8). *Portulaca oleracea* is the most frequently wild edible species found to be used in ten Arab countries, where its leaves are used fresh raw or cooked as recorded in this study (Tables 2, 5). *Ziziphus spina-christi* is the second frequently used plant in eight Arab countries, where its fruits are eaten raw or dried (Tables 2, 5).

Despite the presence of similar linguistic, cultural and ethnic characteristics in Arab countries compared to southern Yemen, there are differences in topography, climate and great geographical distance. This led to significant differences between the flora of Yemen and the flora of Arab countries in northern Arabia and northern Africa.

### Novel WEPs for Yemen

Interestingly, the 58 WEPs recorded in this study have never been reported before from southern Yemen except *Cissus rotundifolia* and *Hydnora abyssinica* which were reported for southern Yemen in our previous studies [23, 34]. For all Yemen regions, there are only three shared species that were reported as WEPs in Soqotra island [6] (Table 2). Even all the recorded local WEPs reported in our study for the first time for inland Yemen, many of them were reported as WEPs in the traditional knowledge of other world countries based on the ethnobotanical literature, with some different in edible parts or consumption modes (Tables 2, 7). Interestingly, the comparative analysis led us to identify twelve new edible plants recorded for the study area (southern Yemen) that have not previously been reported as WEPs in the ethnobotanical literature of other world countries; they are marked with bold in Table 2 and summarized in Table 7. Seven of them belong to Apocynaceae. All of the new recorded edible species are indigenous or endemic to Yemen and Arab peninsula.

### Traditional food security in southern Yemen

The reported WEPs are an important part of the historical, cultural and biological heritage in southern Yemen. This is evidenced through the large number of novel local wild edible species, their widespread use, wide abundance and wide diversity in southern Yemen.

Therefore, these plants have an essential role in the food security of people during food shortage and the military conflicts in Yemen. Furthermore, WEPs provide healthy food and increase the family's income. Moreover, wild edible trees provide shade and wood provides tools for construction, agricultural and kitchen tools, and livestock protection. The most famous local wild edible trees include *Ziziphus spina-christi* and *Acacia nilotica*. Therefore, there is a strong ancient traditional and cultural relationship between tribe members and wild plants. Tribes have the strong control on the protection of their own environment including wild plants through tribal traditional laws which protect wild plants including WEPs. Therefore, the local people usually appreciate the importance of the wild plants for their life and for the healthy environment through the tribal traditions related to plants in the tribal area. Tribal laws, tribal customs and local tribal control on their areas were the most important reasons that preserved the local heritage of the traditional knowledge of WEPs from extinction and indiscriminate uses, despite the absence of the central state due to political, war and military conflicts in the country.

## Conclusions

This is the first and sole ethnobotanical study on the traditional knowledge WEPs from Yemen. The study reports for the first time 58 local WEPs consumed by local people in southern Yemen. Moreover, a comparison with the ethnobotanical literature conducted in world countries led to identify new 12 wild species as new WEPs that have never been reported before. These include the flower of *Aloe lanata* and *Anisotes trisulcus*, stem of *Desmidorchis awdelianus*, *Monolluma hexagona*, *M. solenophora* and *Orbea wissmannii*. This result evidences the strong relationship between the indigenous people and the nature. Nowadays, most local people in southern Yemen still consume WEPs as food security during the war and as local traditional culture. They faith that these local WEPs contain high and specific nutritional values compared to the industrial food and have high potential power to increase the human immunity against diseases. Therefore, many local WEPs become to have commercial values and use frequently including *Amaranthus graecizans, Corchorus tridens, Hydnora abyssinica, Monolluma quadrangula, Portulaca oleracea,* and *Ziziphus spina-christi*. These findings strengthen the importance of the traditional knowledge of the WEPs in southern Yemen to preserve it against risks such wars, conflicts and economic crises that threaten human and local traditional knowledge.

## Data Availability

All data generated or analyzed during this survey are included in this article.

## References

[1] Heinrich M, Kufer J, Leonti M, Pardo-de-Santayana M (2006). Ethnobotany and ethnopharmacology interdisciplinary links with the historical sciences. J Ethnopharmacol.

[2] Schulp CJE, Thuiller W, Verburg PH (2014). Wild food in Europe: A synthesis of knowledge and data of terrestrial wild food as an ecosystem service. Ecol Econ.

[3] Abbasi AM, Khan MA, Khan N, Shah MH (2013). Ethnobotanical survey of medicinally important wild edible fruits species used by tribal communities of Lesser Himalayas-Pakistan. J Ethnopharmacol.

[4] Addis G, Urga K, Dikasso D (2005). Ethnobotanical study of edible wild plants in some selected districts of Ethiopia. Hum Ecol.

[5] Al-Fatimi M (2019). Ethnobotanical survey of medicinal plants in central Abyan governorate. Yemen J Ethnopharmacol.

[6] Miller AG, Morris M. Ethnoflora of the Socotra Archipelago. Royal Botanic Garden Edinburgh; 2004.

[7] Kilian N, Hein P, Hubaishan M.A. (ed.). Further notes on the flora of the southern coastal mountains of Yemen. Willdenowia. 2004;34:59–182. 10.3372/wi.34.34114.

[8] Sanchez-Bel P, Romojaro A, Egea I, Pretel MT (2015). Wild edible plants as potential antioxidant or nutritional supplements for beverages minimally processed. LWT-Food Sci Technol.

[9] Miller, A.G.; Morris, M. Plants of Dhofar, The Southern Region of Oman: Traditional, Economic, and Medicinal Uses. [Muscat]: Office of the Adviser for Conservation of the Environment, Diwan of Royal Court, Sultanate of Oman; 1988.

[10] Phillips GO, Ogasawara T, Ushida K (2008). The regulatory and scientific approach to defining gum arabic (*Acacia senegal* and *Acacia seyal*) as a dietary fibre. Food Hydrocoll.

[11] Fentahun MT, Hager H (2009). Exploiting locally available resources for food and nutritional security enhancement: wild fruits diversity, potential and state of exploitation in the Amhara region of Ethiopia. Food Sec.

[12] FAO/WFP Crop and Food Security Assessment Mission to Yemen. Food and Agriculture Organization of the United Nations, Rome; 2009.

[13] Meve U, Liede S (2002). A molecular phylogeny and generic rearrangement of the stapelioid Ceropegieae (Apocynaceae-Asclepiadoideae). Plant Syst Evol.

[14] Egadu SP, Mucunguzi P, Obua J (2007). Uses of tree species producing gum Arabic in Karamoja, Uganda. Afr J Ecol.

[15] Motlagh S, Ravines P, Karamallah KA, Ma Q (2006). The analysis of *Acacia* gums using electrophoresis. Food Hydrocoll.

[16] Molla EL, Asfaw Z, Kelbessa E, Van Damme P (2011). Wild edible plants in Ethiopia: a review on their potential to combat food insecurity. Afrika Focus..

[17] Aryal KP, Poudel S, Chaudhary RP, Chettri N, Chaudhary P, Ning W, Kotru R (2018). Diversity and use of wild and non-cultivated edible plants in the Western Himalaya. J Ethnobiol Ethnomed.

[18] Urso V, Signorini MA, Tonini M, Bruschi P (2016). Wild medicinal and food plants used by communities living in Mopane woodlands of southern Angola: Results of an ethnobotanical field investigation. J Ethnopharmacol.

[19] Mahklouf M (2019). Ethnobotanical study of edible wild plants in Libya. Eur J Ecol.

[20] Abdel-Sattar EA, Abdallah HM, Khedr A, Abdel-Naim AB, Shehata IA (2013). Antihyperglycemic activity of *Caralluma tuberculata* in streptozotocin-induced diabetic rats. Food Chem Toxicol.

[21] Sadeghi Z, Valizadeh J, Shermeh OA, Akaberi M (2015). Antioxidant activity and total phenolic content of *Boerhavia elegans* (choisy) grown in Baluchestan, Iran. Avicenna J Phytomed.

[22] Selvaraj D, Sarma RK, Shanmughanandhan D, Srinivasan R, Ramalingam S (2015). Evaluation of DNA barcode candidates for the discrimination of the large plant family Apocynaceae. Plant Syst Evol.

[23] Al-Fatimi M, Wurster M, Schröder G, Lindequist U (2007). Antioxidant, antimicrobial and cytotoxic activities of selected medicinal plants from Yemen. J Ethnopharmacol.

[24] Hegazy AK, Mohamed AA, Ali SI, Alghamdi NM, Abdel-Rahman AM, Al-Sobeai S (2019). Chemical ingredients and antioxidant activities of underutilized wild fruits. Heliyon.

[25] Bvenura C, Sivakumar D (2017). The role of wild fruits and vegetables in delivering a balanced and healthy diet. Food Res Int.

[26] Balemie K, Kebebew F (2006). Ethnobotanical study of wild edible plants in Derashe and Kucha Districts, South Ethiopia. J Ethnobiol Ethnomed.

[27] Welcome AK, Van Wyk BE (2019). An inventory and analysis of the food plants of southern Africa. S Afr J Bot.

[28] Ghazanfar SA. Edible wild plants: a case study from Oman. In: Global perspectives on underutilized crops. Springer, Cham; 2018. p. 207–16.

[29] Ojelel S, Mucunguzi P, Katuura E, Kakudidi EK, Namaganda M, Kalema J (2019). Wild edible plants used by communities in and around selected forest reserves of Teso-Karamoja region, Uganda. J Ethnobiol Ethnomed.

[30] Hegazy AK, Al-Rowaily SL, Faisal M, Alatar AA, El-Bana MI, Assaeed AM (2013). Nutritive value and antioxidant activity of some edible wild fruits in the Middle East. J Med Plants Res.

[31] Salih NKEM, Ali AH (2014). Wild food trees in eastern Nuba Mountains, Sudan: use, diversity, and threatening factors. J Agric Rural Dev Trop Subtrop.

[32] Ali-Shtayeh MS, Jamous RM, Al-Shafie’ JH, Elgharabah WA, Kherfan FA, Qarariah KH (2008). Traditional knowledge of wild edible plants used in Palestine (Northern West Bank): A comparative study. J Ethnobiol Ethnomed.

[33] Tebkew M, Gebremariam Y, Mucheye T, Alemu A, Abich A, Fikir D (2018). Uses of wild edible plants in Quara district, northwest Ethiopia: implication for forest management. Agric Food Secur.

[34] Al-Fatimi M, Ali NA, Kilian N, Franke K, Arnold N, Kuhnt C, Schmidt J, Lindequist J (2016). Ethnobotany, chemical constituents and biological activities of the flowers of Hydnora abyssinica A.Br. (Hynoraceae). Pharmazie.

[35] Dogan Y (2012). Traditionally used wild edible greens in the Aegean Region of Turkey. Acta Soc. Bot. Pol. Pol. Tow. Bot..

[36] Zeghad N, Ahmed E, Belkhiri A, Heyden YV, Demeyer K (2019). Antioxidant activity of *Vitis vinifera, Punica granatum, Citrus aurantium* and *Opuntia ficus indica* fruits cultivated in Algeria. Heliyon.

[37] Guil-Guerrero JL, Rodríguez-García I (1999). Lipids classes, fatty acids and carotenes of the leaves of six edible wild plants. Eur Food Res Technol.

[38] Marouf M, Batal M, Moledor S, Talhouk SN (2015). Exploring the practice of traditional wild plant collection in Lebanon. Food Cult Soc.

[39] Hadjichambis ACH, Hadjichambi DP, Della A, Giusti ME, Pasquale DC, Lenzarini C, Censorii E, Tejero MRG, Rojas CPS, Gutierrez JRR, Skoula M, Johnson CH, Sarpakia A, Hmomouchiv M, Jorhi S, Demerdash ME, Zayat M, Pioroni A (2008). Wild and semi-domesticated food plant consumption in seven circum-Mediterranean areas. Int J Food Sci Nut.

[40] Tukan SK, Takruri HR, El-Eisawi DM (1998). The use of wild edible plants in the Jordanian diet. Int J Food Sci Nutr.

[41] Obied W, Mohamoud E, Mohamed O (2003). *Portulaca oleracea* (Purslane): nutritive composition and clinico-pathological effects on Nubian goats. Small Rumin Res.

[42] Al-Asmari AK, Al-Elaiwi AM, Athar MT, Tariq M, Al Eid A, Al-Asmary SMA. review of hepatoprotective plants used in Saudi traditional medicine. Evid. Based Complement. Altern. Med. 2014:890842. 10.1155/2014/890842 PMC4281445 25587347

[43] Samuelsson G, Farah MH, Claeson P, Hagos M, Thulin M, Hedberg O, Warfa AM, Hassan AO, Elmi AH, Abdurahman AD, Elmi AS, Abdi YA, Alin MH (2012). Inventory of plants used in traditional medicine in Somalia. IV. Plants of the families Passifloraceae-Zygophyllaceae. J Ethnopharmacol.

[44] Pieroni A, Zahir H, Amin HIM, Sõukand R (2019). Where tulips and crocuses are popular food snacks: Kurdish traditional foraging reveals traces of mobile pastoralism in Southern Iraqi Kurdistan. J Ethnobiol Ethnomed.

[45] Bruyns PV (1999). The systematic position of *Rhytidocaulon* (Apocynaceae-Asclepiadoideae). Edinb J Bot.

[46] Dop MC, Kefi F, Karous O, Verger EO, Bahrini A (2019). Identification and frequency of consumption of wild edible plants over a year in central Tunisia: a mixed-methods approach. Public Health Nutr.

[47] Aati H, El-Gamal A, Shaheen H, Kayser O (2019). Traditional use of ethnomedicinal native plants in the Kingdom of Saudi Arabia. J Ethnobiol Ethnomed.

[48] Ivanišová E, Grygorieva O, Abrahamová V, Schubertova Z, Terentjeva M, Brindza J (2017). Characterization of morphological parameters and biological activity of jujube fruit (*Ziziphus jujuba* Mill.). J. Berry Res..

[49] El Maaiden E, El Kharrassi Y, Moustaid K, Essamadi AK, Nasser B (2019). Comparative study of phytochemical profile between *Ziziphus spina christi* and *Ziziphus lotus* from Morocco. J Food Meas Charact.

[50] Omino E, Kokwaro J (1993). Ethnobotany of Apocynaceae species in Kenya. J Ethnopharmacol.

[51] Ruiters AK, Gericke N, Stander M, Van Wyk BE (2016). The Apocynaceae as a major source of functional foods in Southern Africa. Planta Med.

[52] Freiberger CE, Vanderjagt DJ, Pastuszyn A, Glew RS, Mounkaila G, Millson M, Glew RH (1998). Nutrient content of the edible leaves of seven wild plants from Niger. Plant Foods Hum Nutr.

[53] Ansari NM, Houlihan L, Hussain B, Pieroni A (2005). Antioxidant activity of five vegetables traditionally consumed by South-Asian migrants in Bradford, Yorkshire, UK. Phytother Res.

[54] Ahmad B, Abbas SJ, Hussain F, Bashir SH, Ahmad D (2014). Study on *Caralluma tuberculata* nutritional composition and its importance as medicinal plant. Pak J Bot.

[55] Teklehaymanot T, Giday M (2010). Ethnobotanical study of wild edible plants of Kara and Kwego semi-pastoralist people in Lower Omo River Valley, Debub Omo Zone, SNNPR, Ethiopia. J Ethnobiol Ethnomed.

[56] Bortolotto IM, de Mello Amorozo MC, Neto GG, Oldeland J, Damasceno-Junior GA (2015). Knowledge and use of wild edible plants in rural communities along Paraguay River, Pantanal, Brazil. J Ethnobiol Ethnomed.

